# Pyrroloquinoline Quinone and NAD^+^ Metabolism in Glaucoma: A Molecular Rationale for Combined Neuroprotection

**DOI:** 10.3390/ph19081268

**Published:** 2026-08-11

**Authors:** Alessandro Medoro, Sergio Davinelli, Cosimo Giuseppe Mazzotta, Luca Agnifili, Giovanni Scapagnini

**Affiliations:** 1 Department of Medicine and Health Sciences “V. Tiberio”, Università degli Studi del Molise, 86100 Campobasso, Italy; alessandro.medoro@unimol.it (A.M.); giovanni.scapagnini@unimol.it (G.S.); 2 Department of Medicine and Surgery, Ophthalmology Unit, Faculty of Medicine and Surgery, University Kore of Enna, 94100 Enna, Italy; cosimogiuseppe.mazzotta@unikore.it; 3 Siena Cross-Linking Center, 53035 Monteriggioni, Italy; 4 Ophthalmology Clinic, Department of Medicine and Ageing Science, University “G. d’Annunzio” of Chieti-Pescara, 66100 Chieti, Italy; luca.agnifili@unich.it

**Keywords:** pyrroloquinoline quinone, NAD^+^ metabolism, aging, age-related disease, glaucoma, retinal ganglion cell, neuroprotection

## Abstract

Glaucoma is the leading cause of irreversible blindness worldwide and a paradigmatic age-related neurodegenerative disease in which retinal ganglion cells (RGCs) are selectively lost through mechanisms that extend beyond intraocular pressure. Age-dependent NAD^+^ depletion in RGCs, compounded by the progressive impairment of NAD^+^ biosynthesis and by the hyperactivation of NAD^+^-consuming enzymes under oxidative stress, defines a metabolic vulnerability that current pressure-lowering therapy does not address. Pyrroloquinoline quinone (PQQ) is a tricyclic ortho-quinone present in plant-derived foods that acts on the NAD^+^ pool through a mechanism distinct from that of conventional precursors. Rather than expanding the pool by net synthesis, PQQ binds lactate dehydrogenase and oxidizes NADH to NAD^+^ through catalytic redox cycling, raising NAD^+^ availability without altering the total dinucleotide content and independently of the two biosynthetic enzymes selectively impaired in glaucomatous RGCs. The resulting increase in NAD^+^ availability activates sirtuin-dependent programs that drive mitochondrial biogenesis. PQQ additionally engages an NRF2-dependent antioxidant response, addressing molecular deficits directly implicated in glaucomatous RGC degeneration. In retinal cell models, PQQ preserves ATP content and viability under mitochondrial stress. In vivo, it protects RGC density in optic nerve degeneration models and elevates NAD^+^ along the visual pathway. A randomized clinical trial demonstrated functional improvement in glaucoma patients receiving a PQQ-containing combination. The redox biochemistry of PQQ places it at a mechanistic intersection with the NAD^+^ deficit that characterizes glaucomatous neurodegeneration. Its complementarity with conventional NAD^+^ precursors and neuroprotective compounds acting on distinct molecular targets supports the design of combination regimens addressing multiple dimensions of RGC vulnerability. Critical questions regarding bioavailability, molecular target characterization, and clinical validation in dedicated trials remain open.

## 1. Introduction

Glaucoma is the leading cause of irreversible age-related blindness worldwide, with global prevalence estimated at 64.3 million in individuals aged 40 to 80 years in 2013 and projected to reach 111.8 million by 2040 [1]. The defining pathological event of glaucoma is the progressive degeneration of retinal ganglion cells (RGCs) and their axons, which together form the optic nerve and transmit visual signals from the retina to the brain. RGCs are post-mitotic neurons of the central nervous system (CNS) with no regenerative capacity in the mammalian retina. Their loss is therefore permanent, and therapeutic strategies can preserve function only in the neurons that remain viable at the time of intervention [2].

The current standard of care addresses glaucoma exclusively through the reduction in intraocular pressure (IOP), the only modifiable risk factor for which long-term randomized evidence of treatment efficacy is available. In the Early Manifest Glaucoma Trial, however, 45% of treated patients showed disease progression despite an average IOP reduction of 25% [3]. A clinical phenotype known as normal-tension glaucoma compounds this limitation, since RGC degeneration in these patients occurs at IOP values within the statistically normal range [2]. Aging is the most significant non-modifiable risk factor for glaucoma, whose prevalence rises steeply after the sixth decade of life; the molecular signature of glaucomatous RGCs recapitulates conserved hallmarks of cellular aging, including mitochondrial dysfunction, genomic instability, and cellular senescence [4,5]. Glaucoma has therefore been reframed as a multifactorial neurodegenerative disease in which the metabolic vulnerability of RGCs operates as an independent and therapeutically actionable mechanism. The energetic demands of RGCs rank among the highest in the CNS. The unmyelinated portion of the RGC axon traverses the optic nerve head and sustains continuous non-saltatory conduction at a metabolic cost met almost exclusively by mitochondrial oxidative phosphorylation, a configuration that underlies the selective vulnerability of RGCs in mitochondrial optic neuropathies such as Leber hereditary optic neuropathy and autosomal dominant optic atrophy [6].

Retinal nicotinamide adenine dinucleotide (NAD^+^) declines in an age-dependent manner, and this depletion precedes and predisposes to the RGC degeneration triggered by elevated IOP [7]. As recently demonstrated in studies involving patients with open-angle glaucoma, mitochondrial dysfunction may represent one of the key mechanisms underlying RGC loss and disease progression. By measuring the oxygen consumption rate in peripheral blood mononuclear cells, basal respiration was found to be lower in patients with high-tension glaucoma and lowest in normal-tension glaucoma relative to age-similar controls, and total cellular NAD was correspondingly reduced from 0.9 pg per mg protein in controls to 0.6 in high-tension and 0.4 in normal-tension glaucoma [8]. This systemic metabolic defect is consistent with the lower plasma nicotinamide (NAM) concentration documented in patients with primary open-angle glaucoma [9]. The causal relevance of the NAD^+−^ dependent mitochondrial pathway is supported by the demonstration that supplementation with the NAD^+^ precursor NAM prevented glaucoma in 93% of eyes at the highest oral dose tested in the DBA/2J mouse model, a widely used model of chronic, age-related, inherited glaucoma [7,10]. The neuroprotective effect of NAD^+^ restoration in animal models has been confirmed across multiple insults, including elevated IOP and optic nerve axotomy [11]. Proof-of-principle evidence in human disease supports NAD^+^ metabolism as a tractable therapeutic target in glaucoma [12,13].

Pyrroloquinoline quinone (PQQ) is an ortho-quinone of dietary origin increasingly recognized as a geroprotective phytochemical. Dietary PQQ supplementation in naturally aged mice attenuates multiple aging phenotypes and reduces oxidative damage across tissues through mechanisms that intersect with mitochondrial dysfunction and deregulated nutrient sensing, two conserved hallmarks of aging [5,14]. PQQ binds mammalian lactate dehydrogenase (LDH) and oxidizes NADH to NAD^+^ through redox cycling, shifting substrate flux from lactate toward pyruvate and increasing intracellular ATP content [15]. In hepatocytes and fibroblasts, PQQ stimulates mitochondrial biogenesis through cAMP response element-binding protein (CREB) and peroxisome proliferator-activated receptor-γ coactivator-1α (PGC-1α), inducing nuclear respiratory factors 1 and 2 (NRF-1 and NRF-2) and mitochondrial transcription factor A (TFAM) [16,17]. The convergence of these two activities places PQQ at a biochemical intersection with the NAD^+^ deficiency that characterizes glaucomatous neurodegeneration, linking this dietary compound to the aging hallmarks that drive optic neuropathy [7,15,16].

This narrative review examines the molecular logic that connects PQQ and NAD^+^ metabolism in the context of retinal neurodegeneration, with primary reference to glaucoma as the paradigmatic optic neuropathy in which this axis is most relevant. The biochemical basis of NAD^+^ homeostasis in the retina, the mechanisms by which its disruption drives RGC death, the redox and mitochondrial biology of PQQ, and the convergent effector pathways through which these two systems interact are addressed in sequence, followed by a stratified evaluation of the available in vitro, preclinical, and clinical evidence and a discussion of the unresolved questions that constrain translational development.

## 2. NAD^+^ Metabolism in the Retina and Its Role in Glaucomatous Neurodegeneration

### 2.1. Biosynthetic Routes and Subcellular Compartmentalization of NAD^+^

NAD^+^ is synthesized in mammalian cells through three biosynthetic routes that converge on a small set of central intermediates [18]. The de novo pathway initiates with tryptophan, which is oxidized by indoleamine 2,3-dioxygenase (IDO) or tryptophan 2,3-dioxygenase (TDO) to *N*-formylkynurenine [19]. Sequential enzymatic steps generate 3-hydroxyanthranilic acid, which is cleaved to α-amino-β-carboxymuconate-ε-semialdehyde by 3-hydroxyanthranilate 3,4-dioxygenase (HAAO). This intermediate cyclizes spontaneously to quinolinic acid unless α-amino-β-carboxymuconate-ε-semialdehyde decarboxylase (ACMSD) diverts it toward picolinic acid. Quinolinic acid is converted to nicotinic acid mononucleotide by quinolinate phosphoribosyltransferase (QPRT), the committed enzyme of the de novo route, which operates predominantly in the liver and is quantitatively minor in most extrahepatic tissues [20]. In the DBA/2J retina, tryptophan, kynurenine, kynurenic acid, and 3-hydroxykynurenine are all reduced relative to control strains, indicating that the de novo route is itself compromised in glaucomatous tissue [21].

The Preiss–Handler pathway uses dietary nicotinic acid and converges with the de novo route at nicotinic acid mononucleotide, produced by nicotinic acid phosphoribosyltransferase (NAPRT). Nicotinic acid mononucleotide derived from either route is adenylated to nicotinic acid adenine dinucleotide by the nicotinamide mononucleotide adenylyltransferase (NMNAT) family, and the product is amidated to NAD^+^ by glutamine-dependent NAD^+^ synthetase. The salvage pathway recycles NAM released by NAD^+^-consuming reactions and is the quantitatively dominant route in most extrahepatic tissues, including the retina [19,22]. NAMPT converts NAM to nicotinamide mononucleotide (NMN), the rate-limiting step of the salvage pathway [23]. NMN is then adenylated to NAD^+^ by NMNAT [18]. Nicotinamide riboside (NR), an alternative precursor, enters this pathway through phosphorylation by nicotinamide riboside kinases 1 and 2 (NRK1, NRK2) [19].

The NMNAT family has three isoforms with distinct subcellular distributions: NMNAT1 is nuclear, NMNAT2 is cytoplasmic and Golgi-associated with a short half-life of approximately 4 h, and NMNAT3 has been reported in mitochondria, although this localization remains debated. This spatial organization sustains three functionally distinct NAD^+^ pools whose concentrations are independently regulated [22,24]. The mitochondrial inner membrane is impermeable to free NAD^+^ and NADH, and the mitochondrial NAD^+^ pool is supplied through the dedicated transporter SLC25A51 [25]. Cytosolic NADH cannot directly enter the matrix and its reducing equivalents are transferred to the matrix by the malate-aspartate and glycerol-3-phosphate shuttles [18,22]. The three biosynthetic routes and their relative tissue distribution are summarized in Table 1.

### 2.2. Competing Consumers: Sirtuins, PARP1, and CD38 in the Regulation of NAD^+^ Availability

NAD^+^ is consumed by three non-redox enzyme classes whose combined activity determines steady-state cellular NAD^+^ concentrations [18]. The sirtuin family comprises seven mammalian deacylases with distinct subcellular localizations: SIRT1 and SIRT6 act in the nucleus, SIRT7 in the nucleolus, SIRT2 in the cytoplasm, and SIRT3, SIRT4, and SIRT5 in the mitochondrial matrix [19,26]. Each catalytic cycle cleaves one NAD^+^ molecule and releases NAM as a competitive feedback inhibitor [18]. SIRT3 is the major mitochondrial deacetylase, interacts with the NDUFA9 subunit of complex I and sustains complex I activity, with mitochondria from Sirt3-null animals showing selective inhibition of this complex [27]. It deacetylates the flavoprotein subunit A of succinate dehydrogenase in complex II, activates superoxide dismutase 2 (SOD2), and regulates enzymes of mitochondrial fatty-acid oxidation [26,28,29]. SIRT1 deacetylates and activates PGC-1α, coordinating mitochondrial biogenesis with NAD^+^ availability [30].

Poly(ADP-ribose) polymerase 1 (PARP1) is the most abundant nuclear NAD^+^-consuming enzyme and is activated by DNA single- and double-strand breaks. A single PARP1 catalytic cycle can polymerize 100 to 200 ADP-ribose units onto target proteins, consuming an equivalent number of NAD^+^ molecules [18,19]. Sustained PARP1 hyperactivation under genotoxic stress depletes the cellular NAD^+^ pool and triggers a caspase-independent form of cell death termed parthanatos, in which poly(ADP-ribose) chains translocate to mitochondria, inducing apoptosis-inducing factor (AIF) release and nuclear translocation [31,32].

CD38 is the principal NAD^+^ glycohydrolase of mammalian cells, catalyzing the hydrolysis of the glycosidic bond between NAM and ADP-ribose, which are irreversibly released as products. Over 90% of the enzymatic activity of CD38 is directed toward NAD^+^ hydrolysis, making it a constitutive degradative enzyme rather than a signaling catalyst [33]. The apparent Km of CD38 for NAD^+^ lies in the low micromolar range, well below physiological intracellular NAD^+^ concentrations, making it constitutively active and particularly destructive when NAD^+^ pools are already depleted by reduced biosynthesis or competing consumption [33]. The expression of CD38 rises progressively in multiple tissues with age, making it the major driver of the systemic NAD^+^ decline observed in older organisms [34]. The competitive consumption of NAD^+^ by sirtuins, PARP1, and CD38 places this metabolite at the intersection of metabolism, DNA repair, and inflammatory signaling [33,35].

### 2.3. In Vivo and Clinical Evidence: From NAMPT and NMNAT2 Downregulation to NAD^+^ Deficit in Glaucoma

The age-dependent decline of retinal NAD^+^ was first demonstrated in vivo in the DBA/2J mouse model of inherited glaucoma: retinal NAD^+^ levels declined with age and with IOP exposure and the deficit preceded overt RGC degeneration. Oral NAM supplementation restored retinal NAD^+^ and prevented glaucoma in 93% of treated eyes at the highest dose tested. The neuroprotective effect was reproduced across distinct insult models by both NAM and intravitreal adeno-associated virus serotype 2 (AAV2)-mediated NMNAT1 gene therapy [7,11]. Transcriptomic profiling of retinal ganglion cells in the same model identified reduced expression of both NAMPT and NMNAT2, indicating a coordinated loss of salvage pathway capacity [7,36]. Tribble et al. further extended the analysis to the metabolomic level, demonstrating that ocular hypertension induces metabolic disruption in retinal and optic nerve tissue, with consistent alterations in α-ketoglutaric acid, creatine/creatinine, homocysteine, and glycerophosphocholine. NAM supplementation prevented this metabolic disruption and simultaneously buffered metabolic stress, increased oxidative phosphorylation, mitochondrial size and motility in glaucomatous RGCs. The reversal of the glaucomatous metabolic signature by NAD^+^ repletion may position metabolomics as a mechanistically informative endpoint for future neuroprotection trials [11].

Translatome profiling of RGCs in a silicone oil-induced ocular hypertension model identified selective downregulation of NMNAT2 in glaucomatous RGCs, with NMNAT1 and NMNAT3 unaffected. RGC-specific overexpression of a long-half-life NMNAT2 mutant via AAV2 intravitreal injection restored NAD^+^ levels in glaucomatous RGCs and optic nerves, and functional and morphological neuroprotection was preserved in both the optic nerve crush model and the ocular hypertension model. The defect is therefore localized to the cytoplasmic adenylyltransferase isoform expressed in RGC axons, consistent with the short half-life of NMNAT2 protein, its enrichment in axons, and its position as the most abundant NMNAT isoform in RGCs [24,37,38].

Patients with primary open-angle glaucoma exhibit a 30% reduction in plasma NAM concentration relative to age-matched controls, a finding that extends the metabolic defect from the retina to the systemic compartment [9]. Oxidative stress is well documented in glaucomatous tissues, and the resulting DNA damage may impose an additional consumptive load through PARP1 activation. The overall pattern of reduced biosynthesis, increased consumption, and impaired compartmentalization defines a coherent NAD^+^ deficit phenotype that explains the metabolic vulnerability of RGCs to IOP-related stress [7,11,32,39,40].

## 3. Pyrroloquinoline Quinone: Redox Biochemistry and Mitochondrial Functions

### 3.1. Chemical Structure and Redox Cycling: The Basis of the Catalytic Versatility of PQQ

PQQ is an aromatic ortho-quinone of plant and microbial origin present in measurable amounts in the human diet. The compound was originally identified in the 1960s as a third bacterial redox cofactor distinct from nicotinamide and flavin coenzymes, and its tricyclic structure was characterized in 1979 [41,42]. PQQ accumulates in soil bacteria, plants, and fermented foods, with the highest concentrations in parsley, kiwi fruit, papaya, fermented soybean products, and human breast milk. Free PQQ content in dietary matrices ranges from 3.7 to 61 ng/g as measured by gas chromatography-mass spectrometry, with more recent liquid chromatography-tandem mass spectrometry analyses confirming concentrations in the ng/g or ng/mL range across a broad panel of foods [43,44,45]. The average human dietary intake of PQQ has been estimated at 0.1–1 mg per day [46].

The molecular structure of PQQ is 4,5-dioxo-4,5-dihydro-1H-pyrrolo[2,3-f]quinoline-2,7,9-tricarboxylic acid, a planar tricyclic system bearing two adjacent carbonyl groups that confer ortho-quinone character [47]. The ortho-quinone moiety undergoes reversible two-electron reduction to the corresponding quinol through a semiquinone radical intermediate, and the redox potential of the PQQ/pyrroloquinoline quinol couple permits catalytic cycling without irreversible degradation. This catalytic stability distinguishes PQQ from stoichiometric antioxidants that are consumed during the scavenging of reactive oxygen species (ROS) and underpins both its function as a prosthetic group in bacterial dehydrogenases and its activity as a regulator of mammalian metabolic enzymes [15,46,47].

The redox cycling that regenerates PQQ from its quinol form proceeds through the reduction of molecular oxygen to superoxide, which dismutates to hydrogen peroxide, and time- and concentration-dependent accumulation of hydrogen peroxide has been measured during the incubation of PQQ with NADH [15]. The net redox effect of PQQ is therefore context-dependent: the same cycling that generates NAD^+^ also produces ROS, while the reduced form scavenges radicals catalytically without being consumed [15,47]. In human trabecular meshwork cells, this mechanism has been proposed to account for the dose-dependent increase in non-mitochondrial oxygen consumption measured under PQQ exposure [48].

### 3.2. Modulation of NAD^+^ Availability by PQQ: LDH and NAMPT

The first identified mammalian protein targets of PQQ were characterized through a proteomics pull-down approach using PQQ-immobilized Sepharose beads in mouse NIH/3T3 fibroblasts, which identified six putative PQQ-binding proteins: LDH-A, pyruvate kinase, triosephosphate isomerase, nucleoside diphosphate kinase B, peroxiredoxin-1, and a translation elongation factor. The focus on LDH-A was motivated by the prior identification of PQQ-dependent dehydrogenases in bacteria of the genus *Gluconobacter*, where PQQ serves as a prosthetic group in membrane-bound LDH, suggesting that the PQQ-LDH interaction may reflect an evolutionarily conserved biochemical relationship [15,49].

Bound PQQ does not function as a classical catalytic cofactor of mammalian LDH. Neither PQQ nor its reduced form served as an alternative cofactor for pyruvate reduction, yet both inhibited lactate formation from pyruvate in the presence of NADH and enhanced pyruvate production from lactate in the presence of NAD^+^. Both effects are attributed to the oxidation of NADH to NAD^+^ by PQQ through its redox cycling, which depletes the hydride donor required for the forward reaction while sustaining the NAD^+^-dependent oxidation of lactate. PQQ retains its redox activity when bound to the enzyme: after dialysis to remove the free compound, PQQ-bound LDH alone oxidized NADH to NAD^+^ and catalyzed the conversion of lactate to pyruvate even in the presence of NADH, whereas intact LDH did not. Docking to the apo structure of human LDH-A positioned PQQ within the NADH-binding pocket, with predicted electrostatic contacts between Arg98 and the 7-carboxyl group and between Arg168 and the 2-carboxyl group of PQQ, both residues being conserved across species. No co-crystal structure of the complex has been reported, and no recognized PQQ-binding motif is present in the LDH-A sequence. Whether PQQ binds the five LDH isoenzymes with different affinity has not been investigated. The enzymatic characterization was performed on rabbit muscle LDH, which is predominantly the M_4_ tetramer corresponding to LDH5 and shares 94% sequence homology with mammalian LDH-A, and no comparative binding data are available for the H-subunit-containing isoenzymes LDH1 to LDH4. Since the relative abundance of these isoenzymes varies across tissues, this gap constrains the prediction of the tissue selectivity of the PQQ–LDH interaction [15].

The kinetic consequence is a quantitative shift in LDH equilibrium. LDH-A normally favors the reduction of pyruvate to lactate, but by lowering the NADH/NAD^+^ ratio PQQ drives the enzyme toward the reverse reaction. In NIH/3T3 fibroblasts exposed to 50 nM PQQ and above in serum- and pyruvate-free medium for 24 h, lactate release into the culture medium decreased significantly to approximately 85% of vehicle controls, and intracellular ATP content increased dose-dependently to approximately 125% of controls, consistent with increased pyruvate availability for acetyl-CoA formation and subsequent entry into the tricarboxylic acid (TCA) cycle and oxidative phosphorylation. The magnitude of these effects is modest, and the reverse reaction also carries a kinetic penalty, since the Km of LDH for lactate is markedly increased by PQQ even as V_max_ rises. NADH oxidized by PQQ does not transfer its reducing equivalents to the respiratory chain and does not contribute to the proton motive force, and this cost is offset by the redirection of pyruvate away from lactate fermentation toward complete oxidation in the TCA cycle, so that the net balance favors ATP production [15]. The NAD^+^-elevating effect of PQQ has also been documented in the human hepatocyte cell line HepG2, where exposure to 10–30 µM PQQ increased the expression, and enzymatic activity of SIRT1 and SIRT3, both NAD^+^-dependent deacetylases whose activation links the redox shift to the transcriptional program of mitochondrial biogenesis discussed in Section 3.3 [50]. At the far lower concentrations of 10–100 nM used in NIH/3T3 fibroblasts, PQQ activated SIRT1 without increasing its protein levels, and the inhibitor EX-527 abolished the response, indicating that the effect may depend on SIRT1 enzymatic activity [17].

The nature of this effect is informative. In HepG2 cells and NIH/3T3 fibroblasts, PQQ raised NAD^+^ levels without altering the total NAD^+^ plus NADH pool, whereas NMN raised both the NAD^+^ level and the total pool in fibroblasts. A transcriptional contribution has been proposed but is not corroborated at the protein level. NAMPT mRNA increases as early as 18 h of PQQ exposure in HepG2 cells and NAD^+^-dependent activity rises at 24 h, yet NAMPT protein abundance is unchanged by PQQ in both HepG2 cells and NIH/3T3 fibroblasts [17,50]. Whether the discrepancy reflects post-transcriptional regulation, an increase in catalytic activity without a change in abundance, or a transcriptional response without functional consequence remains unresolved. Our computational study has proposed a molecular basis compatible with the second possibility. Molecular docking positioned PQQ within the allosteric activation site of NAMPT with a binding free energy of −9.4 kcal/mol, alanine substitution of Tyr188 and Val242 abolished the allosteric contacts, and a 100 ns molecular dynamics simulation confirmed retention of the ligand within the site. Allosteric activation may increase catalytic activity without altering protein abundance, consistent with the immunoblot data, but this prediction awaits experimental validation [51].

### 3.3. PQQ-Driven Mitochondrial Biogenesis: PGC-1α, NRF-1, and TFAM as Convergent Effectors

In vivo evidence for a role of PQQ in mitochondrial homeostasis was provided by dietary studies in weanling mice. Mice fed an amino acid-based diet without PQQ supplementation showed a 20–30% reduction in hepatic mitochondrial content relative to PQQ-supplemented controls (2 mg/kg diet), together with reduced respiratory control ratios and respiratory quotients [52].

The mechanistic basis of this phenotype was characterized in mouse Hepa1-6 hepatocytes. Exposure to PQQ at 10–30 μM for 24–48 h increased citrate synthase activity, cytochrome c oxidase activity, mitochondrial DNA content, and cellular oxygen consumption. PQQ stimulated phosphorylation of CREB at Ser133 and activated the promoter of PGC-1α. Small interfering RNA of either CREB or PGC-1α reduced the mitochondrial response to PQQ. PGC-1α coordinates the transcription of nuclear-encoded mitochondrial genes through interaction with NRF-1 and NRF-2. PQQ treatment induced TFAM, TFB1M, and TFB2M, and protected cells against rotenone, 3-nitropropionic acid, antimycin A, and sodium azide [16].

**Figure 1:**
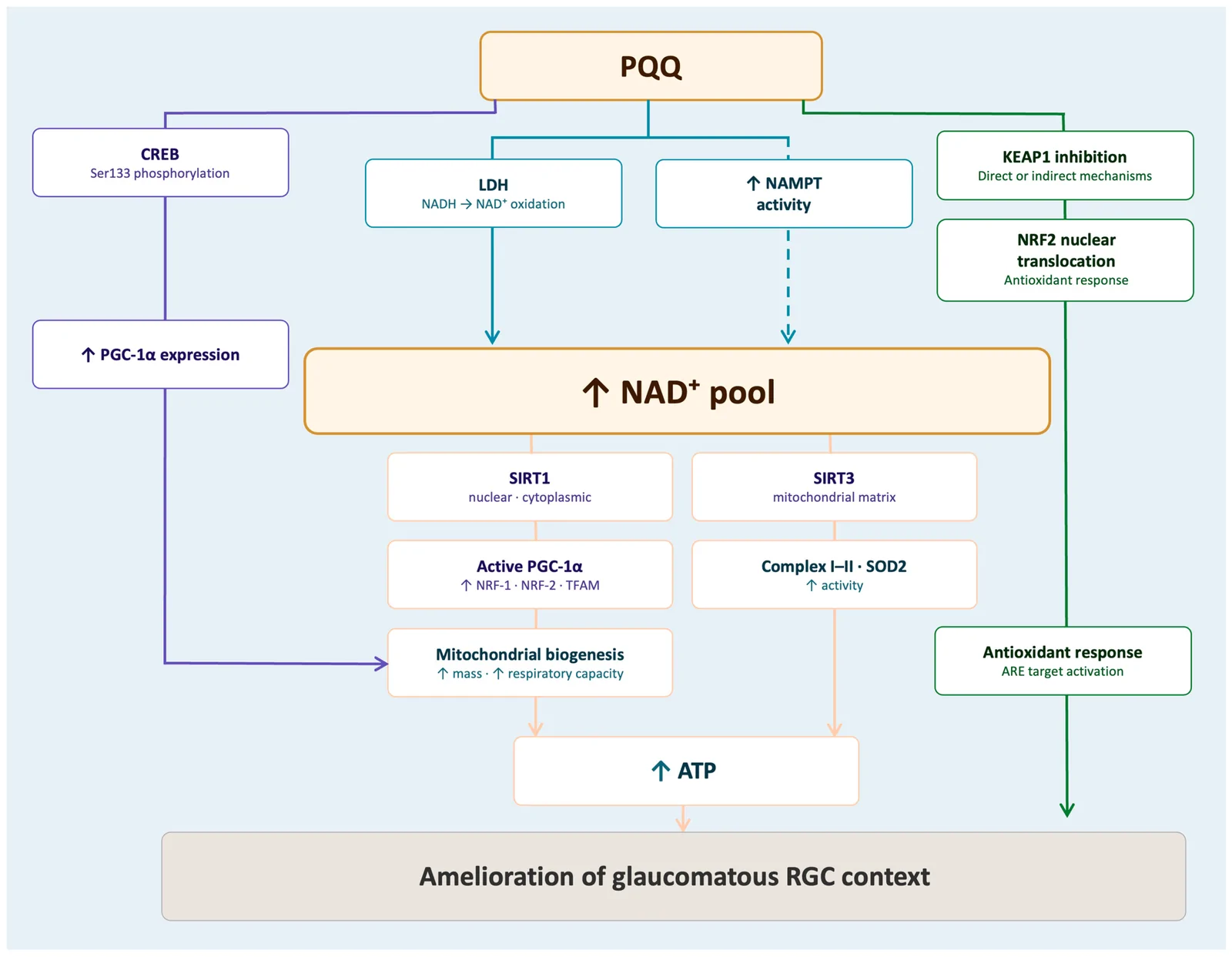
Molecular pathways linking PQQ to NAD^+^ metabolism and mitochondrial function. PQQ raises NAD^+^ availability at lactate dehydrogenase (LDH) by oxidizing NADH and may further increase NAD^+^ through allosteric activation of NAMPT. In parallel, PQQ phosphorylates CREB at Ser133, inducing PGC-1α expression and mitochondrial biogenesis, and engages the KEAP1–NRF2 antioxidant axis. The elevated NAD^+^ pool activates SIRT1 and SIRT3, which deacetylate PGC-1α and respiratory chain components (complexes I and II, SOD2), converging on mitochondrial biogenesis, respiratory capacity and ATP production. In glaucomatous retinal ganglion cells, impaired NAD^+^ homeostasis, mitochondrial dysfunction, and oxidative stress converge to drive degeneration. The pathways shown act on these bioenergetic, biogenic, and antioxidant dimensions to ameliorate RGC vulnerability. Solid arrows denote interactions experimentally demonstrated for PQQ; dashed arrows denote hypothetical or non-corroborated steps.

A subsequent study demonstrated that PQQ activates the SIRT1/PGC-1α signaling axis in NIH/3T3 fibroblasts, producing PGC-1α deacetylation, nuclear translocation, and induction of mitochondrial biogenesis. This finding connects the PQQ-mediated increase in the NAD^+^/NADH ratio, produced at LDH, to the enzymatic activity of SIRT1, which requires NAD^+^ as co-substrate for its deacetylase function (Figure 1) [17].

## 4. The PQQ–NAD^+^ Axis: Convergent Mechanisms in Retinal Ganglion Cell Neuroprotection

### 4.1. Molecular Convergence: NADH Oxidation, Sirtuin Activation, and Biogenic Transcriptional Control

The neuroprotective rationale for PQQ in glaucomatous retinas rests on the molecular intersection between its redox activity and the NAD^+^-dependent machinery that sustains RGC bioenergetics. Across cellular, retinal, and clinical models, the most consistent functional readout of PQQ is the restoration of ATP content under bioenergetic stress (Table 2) [15,48,53]. This convergence on ATP is mechanistically central, because the vulnerability of RGCs in glaucoma is fundamentally energetic, as outlined in the Introduction [6].

PQQ principally acts on the NAD^+^ pool through two complementary mechanisms: an acute enzymatic route via LDH-mediated NADH oxidation, and a second route involving NAMPT, whose transcript increases in hepatocytes without a corresponding change in protein abundance [15,17,50]. We have proposed that this discordance reflects allosteric activation of the enzyme rather than increased expression, a mechanism that would raise catalytic activity at constant protein levels but remains computational and awaits experimental validation [51]. LDH-mediated NADH oxidation increases NAD^+^ availability, which is required as the co-substrate for SIRT1 deacetylase activity. The apparent Km of SIRT1 for NAD^+^ falls within the range of physiological intracellular NAD^+^ concentrations; this proximity to the Km renders SIRT1 a molecular sensor of NAD^+^ availability, such that modest reductions in the NAD^+^ pool produce disproportionate decreases in deacetylase activity [35]. In glaucomatous RGCs, where NAD^+^ declines with age and with IOP exposure, SIRT1 activity is expected to fall well below its half-maximal rate, amplifying the consequences of biosynthetic impairment into broad dysfunction of mitochondrial proteostasis [7,35]. SIRT1 in turn deacetylates and activates PGC-1α, the master regulator of mitochondrial biogenesis, coordinating with NRF-1 and NRF-2 to induce TFAM and expand mitochondrial mass [16,30]. A parallel route involves phosphorylation of CREB at Ser133, which independently activates the PGC-1α promoter [16]. The convergence of acute redox correction and chronic transcriptional reprogramming distinguishes PQQ from supplementation strategies that act on NAD^+^ precursors alone [15,16,17]. The neuroprotective properties of PQQ extend beyond the optic nerve to broader models of neurodegeneration. In an Aβ_1–42_-induced Alzheimer’s disease mouse model, PQQ reduced cognitive deficits and modulated the SIRT1 and CREB pathways, and in a D-galactose-induced aging model, PQQ attenuated cognitive impairment through the protein kinase B (Akt)/glycogen synthase kinase-3β (GSK-3β) signaling pathway [55,56].

In this context, PQQ offers a rationale for intervention at two levels. The PQQ–LDH axis bypasses the impaired NAD^+^ salvage pathway by generating NAD^+^ directly from the existing NADH pool, and the PQQ–SIRT1–PGC-1α axis addresses the downstream consequence of NAD^+^ deficiency, namely the loss of sirtuin-mediated mitochondrial proteostasis (Figure 1) [7,15,17,38].

### 4.2. NRF2/KEAP1 Engagement and Coordinated Antioxidant Response

Oxidative stress contributes substantially to RGC vulnerability in glaucoma, and the NRF2/KEAP1 axis is the principal cellular system that couples redox imbalance to the transcription of cytoprotective genes [2,57]. Under basal conditions, KEAP1 retains NRF2 in the cytoplasm and targets it for proteasomal degradation; oxidative or electrophilic stress modifies cysteine residues on KEAP1 and NRF2 can translocate to the nucleus and activate antioxidant response elements (ARE) [57,58,59].

The mechanism by which PQQ engages this pathway is not settled. In aged mice, dietary PQQ reduced oxidative damage and cellular senescence in nucleus pulposus tissues, and the effect was abolished in NRF2-knockout animals, establishing NRF2 as a non-redundant mediator of its antioxidant action. Docking and co-immunoprecipitation indicated a direct interaction of PQQ with KEAP1 [60]. In aging-related osteoporosis, the same axis was engaged indirectly, through PQQ binding and stabilizing the minichromosome maintenance complex component 3 (MCM3), which then competes with NRF2 for KEAP1 [61]. Whether PQQ acts directly on KEAP1, through an intermediate protein, or both in a tissue-dependent manner remains unresolved, and none of this evidence derives from ocular tissue [60,61].

The integration of NAD^+^ restoration with NRF2 activation may produce a coordinated protective program, since SIRT1 deacetylates NRF2 and modulate its nucleocytoplasmic distribution and transcriptional activity, although the direction of this effect remains debated [62,63]. PQQ may therefore converge on this defensive network at more than one level: it generates NAD^+^ at LDH, activates SIRT1 downstream of NAD^+^, and engages the KEAP1–NRF2 axis, the last through mechanisms so far demonstrated only in non-ocular tissues [15,17,60].

### 4.3. In Vitro Evidence: ATP Rescue and Metabolic Remodeling in Retinal Ganglion Cell Models

PQQ has been directly evaluated in retinal models of mitochondrial stress. In dissociated cortical cells, PQQ increased ATP content dose-dependently from 0.5 µM, reaching more than 50-fold the untreated control at 50 µM, without a corresponding change in NAD content. Targeted metabolomics on PQQ-treated retinal tissue identified shifts in metabolites linked to the TCA cycle and to glycolytic flux, consistent with the redirection of pyruvate toward mitochondrial oxidation predicted by the PQQ–LDH mechanism [53]. The direct binding of PQQ to LDH-A has been shown in fibroblast and in assays with purified enzyme, and its contribution in retinal ganglion cells is supported by the ATP rescue observed in these models rather than by direct measurement of the interaction in retinal tissue [15].

A complementary ex vivo model confirmed PQQ-mediated RGC protection in the native retinal architecture. Mouse retinal explants maintained in culture for three days showed significantly higher density of RNA-binding protein with multiple splicing (RBPMS)-positive RGCs when supplemented with 50–100 µM PQQ relative to vehicle controls, indicating that the effect does not require systemic metabolic input [53]. Mitochondrial biogenesis as a downstream effect of PQQ in retinal tissue was less pronounced than in hepatocyte or fibroblast models. Indeed, in retinal tissue, PQQ increased the complex I subunit NADH:ubiquinone oxidoreductase subunit B8 (NDUFB8) at both transcript and protein level without altering PGC-1α, TFAM, or the mitochondrial-to-nuclear RNA ratio, indicating that biogenic transcriptional control is not the dominant contributor to its effect in this context. The acute redox correction at LDH and the metabolic remodeling appear to be the principal in vitro mechanisms of action of PQQ in RGC-relevant systems [15,53].

### 4.4. Preclinical Evidence: Optic Nerve Protection and IOP-Relevant Cellular Targets

In vivo, intraperitoneal PQQ administration to mice increased ATP content in retina, optic nerve, and superior colliculus, while NAD^+^ increased selectively in the superior colliculus, with no change in the retina or cortex. Administration in drinking water did not reproduce these effects, indicating limited bioavailability by this route [53].

The molecular basis of this selectivity in the NAD^+^ response remains unresolved. One plausible explanation is a differential expression of LDH-A between the retina and the superior colliculus: if LDH-A, the principal PQQ-binding protein, is expressed at higher levels or in a more accessible subcellular configuration in neurons of the superior colliculus than in RGC soma or axonal terminals, PQQ-driven NADH oxidation would generate more NAD^+^ at the post-retinal level than at the retinal level. Alternatively, differences in baseline NADH/NAD^+^ ratios between the two compartments may influence the efficiency of the PQQ–LDH catalytic cycle. Resolving this question has direct implications for dose selection in clinical trials, since the retinal NAD^+^ pool, the primary therapeutic target, may require higher systemic PQQ concentrations or alternative delivery routes to achieve repletion [15,53]. In two independent mouse models of RGC degeneration, ex vivo retinal axotomy and intravitreal rotenone, PQQ administration significantly preserved RGC density and attenuated nuclear shrinkage compared with vehicle-treated controls [53].

The trabecular meshwork represents a parallel cellular target relevant to glaucoma pathogenesis through its control of aqueous humor hydrodynamics and, therefore, IOP [40]. In primary human trabecular meshwork cells, PQQ at 100 nM improved mitochondrial respiration and ATP production, and pretreatment before a hydrogen peroxide challenge normalized proton leak, restored network morphology and cristae architecture, and reduced cell death. These effects occurred without changes in mitochondrial content or in SIRT1 and PGC-1α protein levels, and were attributed to SIRT3 and not to canonical biogenesis [48]. The same dissociation between bioenergetic rescue and biogenic control is seen in retinal tissue, where PQQ raises ATP without inducing the biogenesis machinery, indicating that in both ocular compartments the acute bioenergetic mechanism may predominate over transcriptional reprogramming [15,48,53].

## 5. Clinical Translation and Open Questions

### 5.1. Bioavailability, Pharmacokinetic Considerations and Safety Profile for Ocular Delivery

PQQ is absorbed orally in humans. After a single dose of 0.2 mg/kg, serum concentrations peak at approximately 2 h, and a fraction of the ingested dose is recovered unmodified in the urine Mammalian tissues do not synthesize PQQ de novo, and intracellular accumulation depends on dietary or supplemental intake combined with the trace contribution of gut microbial metabolism. The chemical stability of the o-quinone system permits oral formulation as the disodium salt without loss of redox activity [64,65]. Direct measurements of PQQ concentration in ocular and CNS tissues have not been reported. Evidence of CNS exposure is indirect: intraperitoneal administration in mice raises ATP content in retina, optic nerve, and superior colliculus, and NAD^+^ in the superior colliculus, whereas the brain cortex is unaffected. The proportion of systemic PQQ that crosses the blood-retina barrier and reaches the inner retina has not been directly quantified in human studies, and this gap constrains the design of dose–response trials in patients with optic neuropathies. Administration in drinking water at a nominally equivalent daily dose did not reproduce these effects, indicating insufficient oral bioavailability in this model and leaving open whether dietary supplementation can achieve tissue concentrations comparable to those obtained parenterally [53].

The safety profile of PQQ has been formally assessed. The European Food Safety Authority (EFSA) evaluated PQQ disodium salt as a novel food and concluded that it is safe under the intended conditions of use, based on a no-observed-adverse-effect-level (NOAEL) of 100 mg/kg body weight per day derived from a 90-day repeated-dose oral toxicity study, with a margin of exposure of 344 relative to the maximum proposed intake of 20 mg per day. The critical effect identified in animal studies was the presence of crystals and protein in urine at 200 mg/kg body weight. The authorized use is restricted to healthy adults and excludes pregnant and lactating women, and the same opinion notes that information on the absorption, distribution, metabolism, and excretion of PQQ in animals and in humans remains limited [65]. No dose-finding study has been conducted for any ocular indication, and the doses used in the glaucoma trial and in preclinical models both fall well within the assessed safety margin [53,54].

### 5.2. Clinical Evidence: PQQ-Containing Combinations and Pattern Electroretinogram Outcomes

A multicenter, randomized, single-blind, crossover trial enrolled patients with primary open-angle glaucoma and IOP controlled below 18 mmHg. Patients received either a fixed combination of citicoline 500 mg, homotaurine 50 mg, vitamin B3 54 mg, and PQQ 5 mg, or citicoline 800 mg as a comparator. Both treatments increased P50 and N95 pattern electroretinogram (PERG) amplitudes, functional measures of RGC activity, with a greater effect for the PQQ-containing combination, which alone produced significant improvement in quality-of-life scores, including total score, general health, color vision, and peripheral vision. The result establishes proof of principle for an integrated metabolic intervention but does not constitute monotherapy validation [54].

The broader clinical landscape of NAD^+^-targeted neuroprotection in glaucoma is more advanced. In three short-term, placebo-controlled, prospective, randomized clinical trials involving patients with early-to-moderate high-tension or normal tension glaucoma, oral supplementation with high-dose of NAD^+^ precursor NAM, alone or combined with pyruvate (1.5–3.0 g/day), significantly improved inner retinal electroretinographic parameters and visual field test locations, without major safety concerns. These trials provide the precedent for outcome measures and patient selection criteria that future PQQ monotherapy trials can adopt [12,13,66].

### 5.3. Rational Combination Strategies and Future Trial Design

The mechanistic distinction between PQQ and other neuroprotective compounds supports the design of integrated regimens that exploit non-redundant protective pathways [54]. Conventional precursors and PQQ raise NAD^+^ by distinct mechanisms. NAM, NR, and NMN may increase the pool by net synthesis, and all three converge on NMNAT2, the adenylating enzyme selectively depleted in glaucomatous RGCs, while NAM additionally requires NAMPT. NR and NMN carry a further constraint specific to this cell type: extracellular NMN must first be converted to NR for cellular uptake, and both precursors then depend on NRK1, whose transcripts are expressed at low levels in RGCs [36,67].

PQQ does not increase the total pool. It oxidizes NADH to NAD^+^ at LDH without synthesizing new dinucleotide (Section 3.2), whereas NMN raises both the NAD^+^ level and the total pool [17,50]. Combining a precursor, which restores biosynthetic flux through NMNAT2, with PQQ, which shifts the existing pool toward NAD^+^, could act on both determinants of NAD^+^ availability, a rationale not yet tested experimentally [15,38].

Two additional classes of compounds with established RGC-protective activity converge on this metabolic core through different molecular routes. Compounds acting on membrane phospholipid biosynthesis, of which citicoline (cytidine 5′-diphosphocholine) is the prototype, increase the availability of choline and cytidine for phosphatidylcholine synthesis and protect cultured retinal cells against glutamate excitotoxicity and high-glucose-induced apoptosis [68]. Long-term oral citicoline supplementation in patients with progressing glaucoma and IOP controlled below 18 mmHg reduced the mean rate of visual field progression from −1.1 to −0.15 dB per year over two years. This mechanism is distinct from the NAD^+^-dependent bioenergetic action of PQQ [69,70,71].

γ-aminobutyric acid (GABA)-A receptor modulators of natural origin, of which homotaurine is the most characterized, attenuate glutamate-mediated excitotoxicity through GABAergic inhibitory tone. Homotaurine is a potent GABA-A receptor agonist with an EC50 of 0.4 μM, approximately ten-fold more potent than GABA itself. In rat models of retinal ischemia, intravitreal homotaurine in combination with forskolin and L-carnosine reduced RGC death and was associated with phosphoinositide 3-kinase (PI3K)/Akt pathway upregulation and GSK-3β inhibition [72,73].

The combined action of PQQ-driven mitochondrial protection, NAD^+^ precursor supplementation, phospholipid biosynthesis support, and GABA-A receptor-mediated excitotoxicity control addresses complementary dimensions of RGC vulnerability in glaucoma [7,15,72]. The activation of SIRT1 by PQQ and the supply of NAD^+^ as SIRT1 co-substrate by NAM would be expected to act in a complementary manner, but this combination has not been formally tested in a dedicated glaucoma trial [7,17]. While a fixed PQQ-containing combination has been evaluated clinically, the mechanistic synergy between its individual components has not been isolated, and the additional pairings proposed here on mechanistic grounds, such as PQQ with a NAD^+^ precursor, remain to be tested experimentally [54].

Several open questions constrain translational development. The retinal effect of PQQ on NAD^+^ levels in vivo is tissue-specific and not uniformly distributed across the visual system, with the molecular basis of this regional selectivity still unknown [53]. The structural basis for PQQ binding to LDH-A remains undefined. No experimentally determined structure of the PQQ–LDH complex is available, and the absence of a recognized PQQ-binding motif in the LDH-A sequence limits rational pharmacological optimization of this interaction. The isoenzyme selectivity of this interaction is equally undefined, and comparative binding data across LDH1 to LDH5 would clarify whether the tissue distribution of the M subunit contributes to the regional pattern of the metabolic effects of PQQ [15]. The improvements in PERG amplitude observed after short-term treatment with the combination require confirmation in longer-term studies incorporating both functional and structural endpoints. From a clinical perspective, the neuroprotective efficacy of molecular compounds supported by a strong biological rationale should ultimately be demonstrated by a reduced rate of glaucomatous progression. Such evidence should primarily be sought through standard automated perimetry and, secondarily, through optical coherence tomography measurements of retinal nerve fiber layer and ganglion cell layer thickness in patients receiving supplementation [54]. Direct quantification of NAD^+^ and of NAD^+^-consuming enzyme activity in aqueous humor of treated patients, although technically demanding, would provide the biomarker bridge between the molecular rationale and the clinical outcome [7,11,54].

## 6. Conclusions

The evidence reviewed here identifies PQQ as a mechanistically distinctive neuroprotective agent whose primary mode of action, enzymatic NAD^+^ generation through LDH-mediated NADH oxidation, is independent of the two biosynthetic steps selectively impaired in glaucomatous RGCs, namely NAMPT downregulation and NMNAT2 depletion. This bypass mechanism places PQQ in a complementary rather than redundant relationship with salvage-pathway precursors such as NAM, whose efficacy is contingent on the enzymatic integrity that glaucomatous RGCs progressively lose. Downstream of the NAD^+^ it generates, PQQ activates SIRT1-dependent mitochondrial biogenesis, and it may additionally engage the KEAP1–NRF2 antioxidant axis, an arm so far demonstrated only in non-ocular tissues. PQQ thus addresses the bioenergetic deficit of glaucomatous RGCs through a mechanism that conventional precursors do not reproduce.

Critical gaps remain. The absence of structural data on the PQQ–LDH complex prevents rational pharmacological optimization of this interaction. Retinal bioavailability of orally administered PQQ in humans has not been quantified, and the regional selectivity of the metabolic effects of PQQ in current mouse data raises unresolved questions about dose adequacy for the primary therapeutic target. The single available clinical trial does not permit isolation of the individual contribution of PQQ within a fixed combination, and no biomarker of retinal NAD^+^ status has been validated for use as a trial endpoint in optic neuropathy.

The most pressing translational priority is a dedicated monotherapy trial in which metabolomic of accessible compartments, such as plasma and aqueous humor, applied before and after NAD^+^-targeted supplementation would provide the tissue-level biomarker evidence needed to design adequately powered phase 2 studies. Existing clinical evidence for NAD^+^ restoration, although encouraging, is constrained by short follow-up periods and high-dose supplementation regimens. Whether metabolic intervention can reduce the rate of glaucomatous progression remains to be established using standard automated perimetry supported by structural optical coherence tomography of the retinal nerve fiber layer and ganglion cell layer. The convergence of the PQQ–LDH bypass mechanism with the established neuroprotective rationale for citicoline and homotaurine on complementary molecular targets defines a rational direction for future experimental work rather than a therapeutic recommendation. Whether combination regimens addressing bioenergetic, membrane, and excitotoxic dimensions of RGC vulnerability simultaneously confer additive benefit requires formal evaluation in dedicated trials before any clinical inference can be drawn.

## Figures and Tables

**Table 1:** The NAD^+^ biosynthetic pathways: substrates, rate-limiting enzymes, tissue distribution, and relevance to glaucomatous neurodegeneration.

Pathway	Initial Substrate	Rate-Limiting Enzyme	Convergence Intermediate	Main Tissue Distribution	Relevance in Retina/Glaucoma
De novo	Tryptophan	QPRT	Nicotinic acid mononucleotide	Liver, kidney, immune cells; quantitatively minor in neurons	Tryptophan pathway alterations documented in DBA/2J retina; ACMSD activity controls flux toward NAD^+^ vs. picolinate
Preiss–Handler	Nicotinic acid (dietary)	NAPRT	Nicotinic acid mononucleotide	Liver, kidney, gut; limited in RGCs	Dietary nicotinic acid provides partial compensation; not the primary route in retinal tissue
Salvage	Nicotinamide (NAM); nicotinamide riboside (NR, via NRK1/2)	NAMPT	NMN	Ubiquitous; dominant in retina and neurons	NAMPT downregulated in glaucomatous RGCs; NMNAT2 (cytoplasmic isoform) selectively depleted; primary therapeutic target for NAD^+^ repletion

ACMSD, α-amino-β-carboxymuconate-ε-semialdehyde decarboxylase; DBA/2J, dilute brown non-agouti mouse strain 2J (hereditary glaucoma model); NAD^+^, nicotinamide adenine dinucleotide; NAM, nicotinamide; NAMPT, nicotinamide phosphoribosyltransferase; NAPRT, nicotinic acid phosphoribosyltransferase; NMN, nicotinamide mononucleotide; NMNAT2, nicotinamide mononucleotide adenylyltransferase 2; NR, nicotinamide riboside; NRK1/2, nicotinamide riboside kinases 1 and 2; QPRT, quinolinate phosphoribosyltransferase; RGC, retinal ganglion cell.

**Table 2:** Summary of experimental evidence on the effects of PQQ on NAD^+^ metabolism, mitochondrial function, and retinal ganglion cell survival, organized by level of evidence (in vitro, preclinical, and clinical).

Evidence Level	Model/Study	Dose and Duration	Primary Endpoint	Key Findings	Main Limitations	References
In vitro	Mouse NIH/3T3 fibroblasts	50 nM, for 24 h	Intracellular NAD^+^/NADH ratio; intracellular ATP content	PQQ binds LDH-A, oxidizes NADH to NAD^+^, reduces lactate release, and increases intracellular ATP content	Non-neuronal cell line; no data in RGCs	[15]
In vitro	Mouse Hepa1-6 hepatocytes	10–30 µM for 24–48 h	Citrate synthase and cytochrome c oxidase activity; mtDNA content; oxygen consumption rate; CREB phosphorylation; PGC-1α promoter activity	PQQ activates CREB-dependent PGC-1α expression, inducing mitochondrial biogenesis via NRF-1, NRF-2, and TFAM	Non-neuronal cell line; biogenic response not reproduced in ocular tissue	[16]
In vitro	Human HepG2 cells; mouse NIH/3T3 fibroblasts	10–100 nM for 48 h (NIH/3T3) and 50–100 nM for 6 h (HepG2)	NAD^+^ and total NAD^+^+NADH pool; NAMPT protein; SIRT1-dependent PGC-1α deacetylation and nuclear translocation	PQQ raises NAD^+^ without changing the total pool and activates SIRT1 without increasing SIRT1 or NAMPT protein; EX-527 abolishes the biogenic response	Non-neuronal cell lines; no data in RGCs	[17]
In vitro	Primary human trabecular meshwork cells; H_2_O_2_ oxidative stress	100 nM, for 23 h + 1 h H_2_O_2_	Mitochondrial oxygen consumption rate; mitochondrial network morphology; oxidative damage markers	PQQ increases basal and ATP-linked respiration; pretreatment before H_2_O_2_ reduces proton leak, increases ATP, restores cristae and network morphology, and reduces cell death; mitochondrial content and SIRT1, PGC-1α, SOD2 protein levels are unchanged; effect attributed to SIRT3	Anterior segment; does not address RGC survival; SIRT3 inferred from protein levels, not activity	[48]
In vitro	Dissociated mouse cortical, retinal, and optic nerve cells	0.1–50 µM for 2 h	Intracellular ATP and NAD content; mitochondrial membrane potential; respiratory complex and citrate synthase activity	PQQ increases ATP dose-dependently from 0.5 µM in cortical cells and at 50 µM in retinal cells and optic nerve, without changing NAD content or the activity of complexes II–IV and citrate synthase	Acute incubation in dissociated cells; not RGC-specific; the ATP increase is not accompanied by NAD elevation	[53]
Preclinical	Ex vivo retinal axotomy (explants); intravitreal rotenone model	50–100 µM in culture medium for 3 days (explants); 20 mg/kg intraperitoneally (rotenone model); 20 mg/kg/day in drinking water (bioavailability comparison)	RGC density; nuclear diameter; retina, optic nerve and superior colliculus NAD^+^ and ATP content	PQQ preserves RGC density and attenuates nuclear shrinkage in both models; intraperitoneal administration raises ATP in retina, optic nerve, and superior colliculus, whereas NAD^+^ rises only in the superior colliculus	Acute injury models rather than chronic IOP-driven glaucoma; oral administration ineffective; regional selectivity of the NAD^+^ response unexplained	[53]
Clinical	POAG patients with controlled IOP (<18 mmHg); multicenter randomized crossover trial	PQQ 5 mg/day in fixed combination for 4 months per arm (crossover)	PERG P50 and N95 amplitude; NEI-VFQ25 quality-of-life scores (general health, color vision, peripheral vision)	Both arms increased P50 and N95 amplitudes, with a greater effect for the PQQ-containing combination, which also improved total, general health, color vision, and peripheral vision scores	Fixed combination; PQQ contribution not isolable; short follow-up; functional endpoint only	[54]

ATP, adenosine triphosphate; CREB, cAMP response element-binding protein; IOP, intraocular pressure; LDH-A, lactate dehydrogenase A chain; mtDNA, mitochondrial DNA; NAD^+^, nicotinamide adenine dinucleotide; NADH, reduced nicotinamide adenine dinucleotide; NRF-1, nuclear respiratory factor 1; NRF-2, nuclear respiratory factor 2; PERG, pattern electroretinogram; PGC-1α, peroxisome proliferator-activated receptor-γ coactivator-1α; POAG, primary open-angle glaucoma; PQQ, pyrroloquinoline quinone; RGC, retinal ganglion cell; SIRT1, sirtuin 1; SIRT3, sirtuin 3; SOD2, superoxide dismutase 2; TFAM, mitochondrial transcription factor A.

## Data Availability

No new data were created or analyzed in this study. Data sharing is not applicable to this article.
